# Harmonizing model organism data in the Alliance of Genome Resources

**DOI:** 10.1093/genetics/iyac022

**Published:** 2022-02-25

**Authors:** Alliance of Genome Resources Consortium, Julie Agapite, Laurent-Philippe Albou, Suzanne A Aleksander, Micheal Alexander, Anna V Anagnostopoulos, Giulia Antonazzo, Joanna Argasinska, Valerio Arnaboldi, Helen Attrill, Andrés Becerra, Susan M Bello, Judith A Blake, Olin Blodgett, Yvonne M Bradford, Carol J Bult, Scott Cain, Brian R Calvi, Seth Carbon, Juancarlos Chan, Wen J Chen, J Michael Cherry, Jaehyoung Cho, Karen R Christie, Madeline A Crosby, Paul Davis, Eduardo da Veiga Beltrame, Jeffrey L De Pons, Peter D’Eustachio, Stavros Diamantakis, Mary E Dolan, Gilberto dos Santos, Eric Douglass, Barbara Dunn, Anne Eagle, Dustin Ebert, Stacia R Engel, David Fashena, Saoirse Foley, Ken Frazer, Sibyl Gao, Adam C Gibson, Felix Gondwe, Josh Goodman, L Sian Gramates, Christian A Grove, Paul Hale, Todd Harris, G Thomas Hayman, David P Hill, Douglas G Howe, Kevin L Howe, Yanhui Hu, Sagar Jha, James A Kadin, Thomas C Kaufman, Patrick Kalita, Kalpana Karra, Ranjana Kishore, Anne E Kwitek, Stanley J F Laulederkind, Raymond Lee, Ian Longden, Manuel Luypaert, Kevin A MacPherson, Ryan Martin, Steven J Marygold, Beverley Matthews, Monica S McAndrews, Gillian Millburn, Stuart Miyasato, Howie Motenko, Sierra Moxon, Hans-Michael Muller, Christopher J Mungall, Anushya Muruganujan, Tremayne Mushayahama, Harika S Nalabolu, Robert S Nash, Patrick Ng, Paulo Nuin, Holly Paddock, Michael Paulini, Norbert Perrimon, Christian Pich, Mark Quinton-Tulloch, Daniela Raciti, Sridhar Ramachandran, Joel E Richardson, Susan Russo Gelbart, Leyla Ruzicka, Kevin Schaper, Gary Schindelman, Mary Shimoyama, Matt Simison, David R Shaw, Ajay Shrivatsav, Amy Singer, Marek Skrzypek, Constance M Smith, Cynthia L Smith, Jennifer R Smith, Lincoln Stein, Paul W Sternberg, Christopher J Tabone, Paul D Thomas, Ketaki Thorat, Jyothi Thota, Sabrina Toro, Monika Tomczuk, Vitor Trovisco, Marek A Tutaj, Monika Tutaj, Jose-Maria Urbano, Kimberly Van Auken, Ceri E Van Slyke, Qinghua Wang, Shur-Jen Wang, Shuai Weng, Monte Westerfield, Gary Williams, Laurens G Wilming, Edith D Wong, Adam Wright, Karen Yook, Magdalena Zarowiecki, Pinglei Zhou, Mark Zytkovicz

**Affiliations:** Caltech—Division of Biology and Biological Engineering 140-18, California Institute of Technology, Pasadena, CA 91125, USA

**Keywords:** genome, knowledgebase, phenotype, data mining, biocuration, gene function, gene expression, gene interaction, variants

## Abstract

The Alliance of Genome Resources (the Alliance) is a combined effort of 7 knowledgebase projects: *Saccharomyces* Genome Database, WormBase, FlyBase, Mouse Genome Database, the Zebrafish Information Network, Rat Genome Database, and the Gene Ontology Resource. The Alliance seeks to provide several benefits: better service to the various communities served by these projects; a harmonized view of data for all biomedical researchers, bioinformaticians, clinicians, and students; and a more sustainable infrastructure. The Alliance has harmonized cross-organism data to provide useful comparative views of gene function, gene expression, and human disease relevance. The basis of the comparative views is shared calls of orthology relationships and the use of common ontologies. The key types of data are alleles and variants, gene function based on gene ontology annotations, phenotypes, association to human disease, gene expression, protein–protein and genetic interactions, and participation in pathways. The information is presented on uniform gene pages that allow facile summarization of information about each gene in each of the 7 organisms covered (budding yeast, roundworm *Caenorhabditis elegans*, fruit fly, house mouse, zebrafish, brown rat, and human). The harmonized knowledge is freely available on the alliancegenome.org portal, as downloadable files, and by APIs. We expect other existing and emerging knowledge bases to join in the effort to provide the union of useful data and features that each knowledge base currently provides.

## Introduction: The model organism databases, the goals, and the approach 

### Model organism databases

Over 20 years ago, databases were constructed and then funded for a majority of the intensively studied model organisms. These databases (perhaps more properly called knowledge bases) grew from the curation of information about genes (e.g. the “Red Book;” for *Drosophila melanogaster*; Lindsley and Grell 1968) or software to support genome projects (e.g. ACeDB for the *Caenorhabditis elegans* genome; Martinelli *et al.* 1997). These include the *Saccharomyces* Genome Database (SGD, https://www.yeastgenome.org [accessed 2022 Jan 16]; Engel *et al.* 2021), FlyBase (https://flybase.org [accessed 2022 Jan 16]; Gramates *et al.* 2022), WormBase (https://wormbase.org [accessed 2022 Jan 16]; Davis *et al.* 2022), Mouse Genome Informatics (MGI, http://www.informatics.jax.org/ [accessed 2022 Jan 16]; Ringwald *et al.* 2021), Rat Genome Database (RGD, https://rgd.mcw.edu/ [accessed 2022 Jan 16]; Smith *et al.* 2020, Kaldunski *et al.* 2021), the Zebrafish Information Network (ZFIN, https://zfin.org [accessed 2022 Jan 16]; Bradford *et al.* 2022), PomBase (https://pombase.org [accessed 2022 Jan 16], Harris *et al.* 2021), The Arabidopsis Information Resource (TAIR, https://arabidopsis.org [accessed 2022 Jan 16]; Berardini *et al.* 2015), and Xenbase (xenbase.org [accessed 2022 Jan 16], Fortriede *et al.* 2020). These model organism databases (MODs), expanded in depth and scope as genomics and genome-scale experiments rose in prominence in the research community. Key use cases were curation of gene structure models, systematic mapping of identifiers (IDs), extracting large datasets from supplemental files, and accreting small-scale experiments into large datasets. 

Much of biocuration involves connecting entities (such as genes, proteins, ncRNAs, sequences, chemicals, cells) to each other using controlled vocabularies. Led by the Gene Ontology Consortium (GO; Ashburner *et al.* 2000; Gene Ontology Consortium 2021), descriptions progressed from controlled vocabularies to ontologies, defined set of terms with defined relations that allow information to be structured and thus computable (meaning able to be used in computational analyses). A large swath of information has been organized into ontologies, including evidence, phenotypes, anatomy, life stages, and the relations, themselves. Some of these ontologies are general, like the GO, whereas others are clade-specific.

In the Alliance, the GO ontologies are used for annotation to molecular functions, biological processes, and cellular components (https://geneontology.org); Chemical Entities of Biological Interest (ChEBI; Hastings *et al.* 2016) is used for chemical entities; Evidence & Conclusion Ontology for evidence types (ECO; Giglio *et al.* 2019); Ontology of bioscientific data analysis and data management (EDAM) for metadata (Ison *et al.* 2013); Experimental Factor Ontology for experimental variables (EFO; Malone *et al.* 2010); Human Phenotype Ontology for human phenotypes (HPO; Köhler *et al.* 2021); Mammalian Phenotype ontology for mouse and rat phenotypes (MP; Smith and Eppig 2009); WBPhenotype for worm phenotypes (Schindelman *et al.* 2011); *Drosophila* Phenotype Ontology for fly phenotypes (DPO; Osumi-Sutherland *et al.* 2013); Ascomycete Phenotype Ontology for yeast phenotypes (APO; Engel *et al.* 2010); Proteomics Standards Initiative—Molecular Interaction for molecular interactions (PSI-MI; Kerrien *et al.* 2007), Proteomics Standards Initiative—Protein Modification Ontology for protein modifications (PSI-MOD; Montecchi-Palazzi *et al.* 2008); Disease Ontology for human disease and disease model annotations (DO; Schriml *et al.* 2022); the Cell Ontology for cell type (CL; Diehl *et al.* 2016); Uberon for animal anatomy (Haendel *et al.* 2014); Mouse Developmental Anatomy Ontology for mouse anatomy (EMAPA; Hayamizu *et al.* 2015); Zebrafish anatomy (ZFA) and development ontology for (ZFA; Van Slyke *et al.* 2014); *Drosophila* gross anatomy for fly anatomy (FBbt; Costa *et al.* 2013); *C. elegans* Gross Anatomy Ontology for worm anatomy (WBbt; Lee and Sternberg 2003); WormBase life stage ontology for worm developmental stages (WBls; W. Chen and D. Raciti, unpublished); Sequence Ontology (SO; Mungall *et al.* 2011; Sant *et al.* 2021) for sequence features; Relation Ontology (RO; Smith *et al.* 2005) for relations; and Measurement Method Ontology (MMO; Smith *et al.* 2013) for expression assays.

### Goals of the Alliance of Genome Resources (the Alliance)

The Alliance was formed in 2016 by 6 MODs and the GO Resource to address several problems facing the MODs (Alliance of Genome Resources Consortium 2019, 2020). First, there was a strong need for harmonization, the process of making related information cross-compatible. Researchers want and often need to compute across organisms, and harmonization enables this. For example, for multispecies, multiomic data integration (reviewed by Zhong and Sternberg 2007) there is significant effort necessary to bring all annotations into one place and to devise custom metrics for each type of information. Another aspect is the ease of use; a researcher wants to look at orthologous genes, and although the individual MOD websites provide much of which is superficially common, different MODs use a vastly different look and feel, structure, terminology, and user interface (UI).

Second, the MODs faced issues of sustainability. This issue was highlighted by imminent funding cuts, inflation with flat budgets, and a steady increase in the amount and complexity of data generated by researchers that is appropriate for curation and inclusion in knowledge bases. For example, new methods generate new or significantly greater quantities of data. CRISPR gene editing enables more rapid generation of mutants of all types, and thus more phenotype and other information. Whole-genome sequencing supports molecular identification of natural or induced variants; single-cell RNA sequencing (scRNA-seq) vastly increases cell-by-cell gene expression data (e.g. Taylor *et al.* 2021).

Last, there were promises based on economies of scale and the need for more software development. If several groups develop software that essentially is redundant, but applied to different organisms, there is an opportunity cost; this cost is paid by researchers who want more facile tools. The economies of scale are realized in software maintenance; keeping 6 complex websites up 24/7/365 takes attention and energy. A potentially more complex website serving the functions of many organisms will likely take a fraction of the maintenance effort, and we can adopt Agile and scrum methodologies to software development, bringing new functionality to researchers more swiftly. The Alliance deposits scripts in a publicly accessible Git repository (https://github.com/alliance-genome [accessed 2022 Jan 16]) providing transparency and dissemination of developed software among the genomics community.

### Approaches/philosophy

The Alliance has adopted several key tenets to guide its scope and implementation.

#### Two ways good; 6 ways bad

One challenge is the diversity of opinions about the best way to do things, be it display data, curate papers, or develop software. Each resource and each individual has preferences for how they like to see data—tables vs figures or bar charts vs scatter plots. Moreover, there are typically several ways to do any analysis. We think that 1 or 2 versions of anything should suffice, and thus we seek to reduce the number of pipelines, computation methods, displays, and so forth, to about 2 rather than multiple versions where many are quite similar.

#### The union and the intersection

Our goal is to serve our communities even better than they have been served. We think this is possible because the total of the features (the Union) of the MODs is greater than any existing MOD. To move this project forward, we started with the overlap of features and data (the Intersection).

#### Be modular, flexible, extensible, FAIR

In general, there is a tension between flexibility and performance as evidenced by evolution and engineering. Because we seek to avoid disruption of services to the genetics community (negative selection) but make major changes in infrastructure (evolutionary novelty), we are optimizing this tension. In practice, this can be achieved by performant modules that can be reused as the architecture changes. We adhere to the FAIR principles of being Findable, Accessible, Interoperable, and Reusable (Wilkinson *et al.* 2016).

#### Harmonized data are most useful

Crucial to sustainability and ease of use (especially for one-stop shopping) is harmonization of data where possible. One might naively think that fish and fly anatomies are too different to compare (fins vs wings), but they each have relatively defined anatomy. The harmonization of comparative anatomy is in some cases a research problem, but ontologies can capture current understanding and even multiple hypotheses about homology and analogy (for recent examples and discussion see Gąsiorowski *et al.* 2021; Musser *et al.* 2021; Tarashansky *et al.* 2021). The increased use and curation of standardized ontologies further supports cross-organism searches and insights.

### Harmonized view of MOD data

**Fig. 1.**
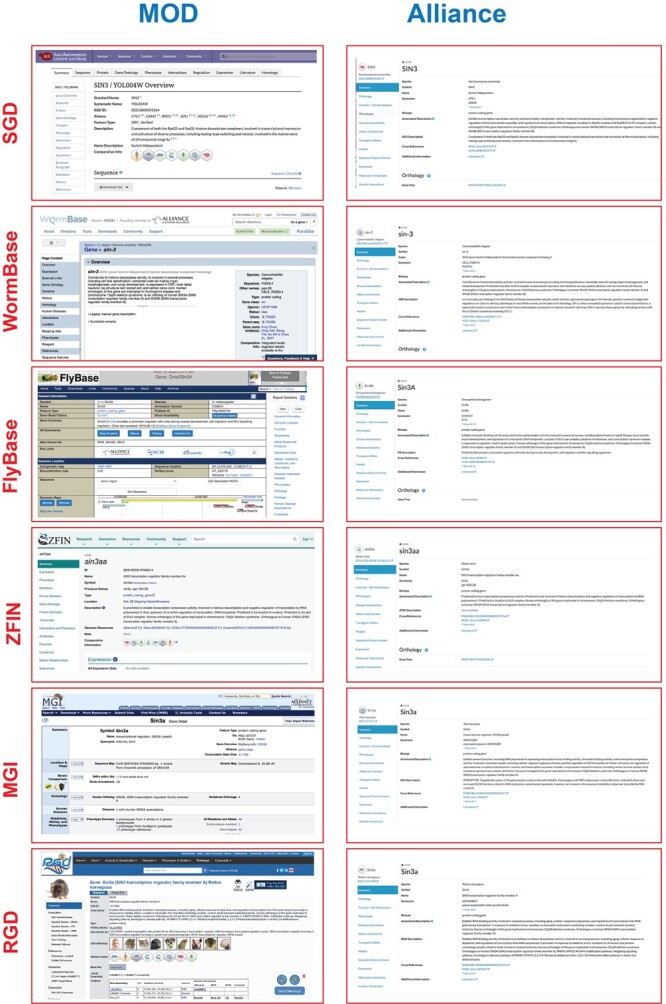
The Alliance Portal provides a harmonized view of research organism information. Left, current MOD pages; Right, current Alliance release 4.0 gene pages.

The Alliance Internet presence (alliancegenome.org [accessed 2022 Jan 16]) provides a consistent view of harmonized information and is laying the foundation to present a harmonized view of un-harmonized information thus capturing the full range of information present in the existing MODs. Figure 1 shows current MOD gene pages and the corresponding Alliance gene page. Although the Alliance pages do not yet have all the information of the MOD gene pages, they provide a consistent view. Moreover, the Alliance provides comparative information that makes use of the harmonized information, as described below.

### Genomes

**Fig. 2.**
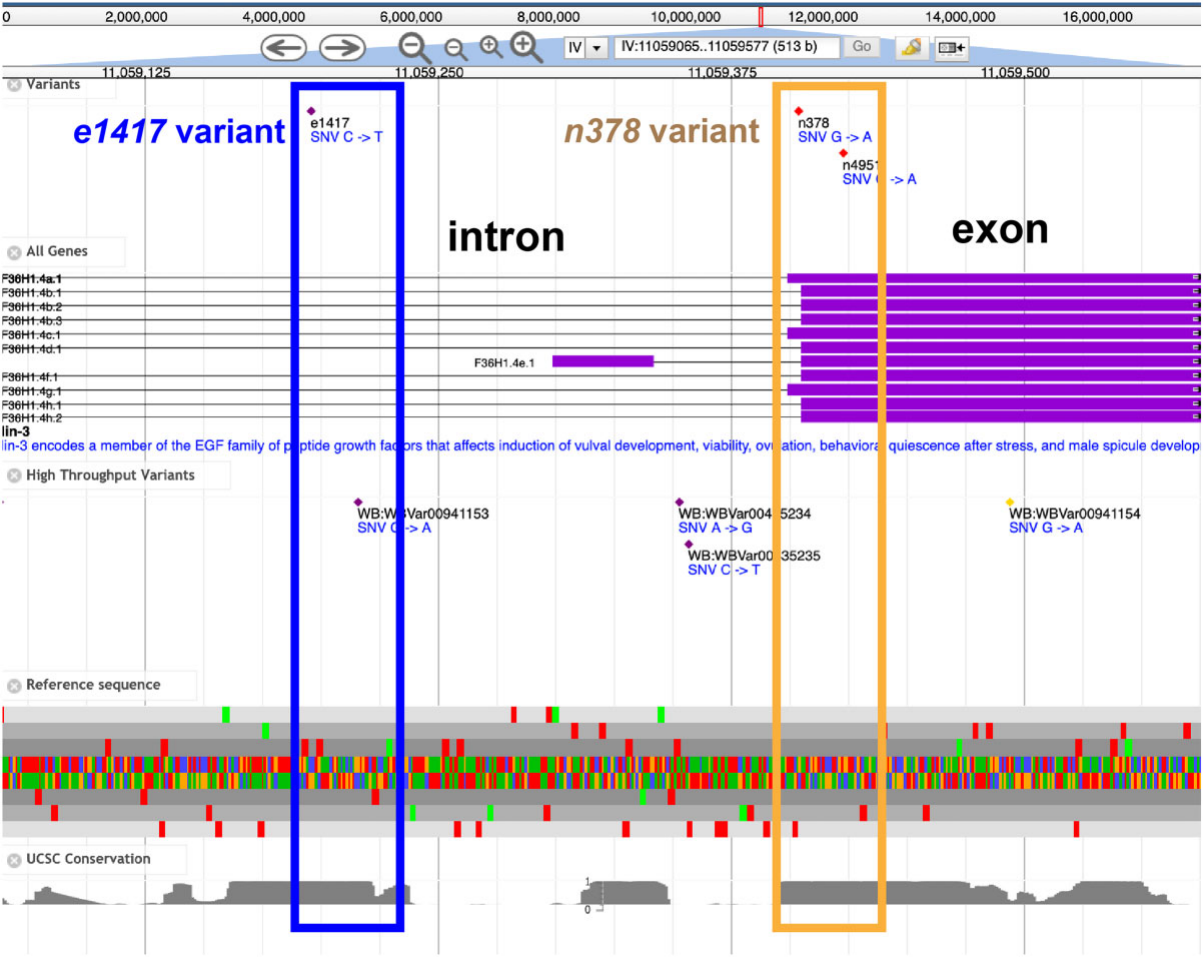
Example of Alliance JBrowse. The top is the standard control bar. Next are curated variants, often with known phenotypic consequences. The gene structure models (introns and exons) for each gene are shown, with the high throughput variants shown in the next track. The reference sequence in all reading frames is followed by a conservation track from University of California, Santa Cruz. Two alleles are highlighted in this figure: the blue box shows the *e1417* allele to be in a conserved intron region, while the gold box shows the *n378* allele to be in a coding exon.

Although the Alliance does not yet support genome annotation per se, it does contain current genome assemblies displayed in a common genome browser (Fig. 2).

Each gene page includes a Genome Features display that has a snapshot of the gene in its genomic context. We recently added more options for initialization of the Browser, which allows us to more flexibility configure the features shown. Such configuration supports interaction with the variants table, providing highlighting and filtering. We also added support for the display of the SARS-CoV-2 genome in addition to the MOD genomes. Moreover, all of the JBrowse (Buels *et al.* 2016) instances at the Alliance now display high throughput variants in a separate track. Furthermore, for nonhuman genomes, another track shows alleles that comprise multiple variants. Finally, there have been bug fixes to support visualizing special characters in FlyBase genes and variants and to support issues related to duplicate naming of transcripts.

We implemented Docker-based solutions for several of the member MODs for their JBrowse services. WormBase’s JBrowse instance has made the most progress, with both its development and production instances of JBrowse now served from Alliance hardware, and a tool for running the JBrowse data production pipeline for WormBase is nearing completion. A new JBrowse instance and data production pipeline was also created for ZFIN with the goal of replacing their aging GBrowse instance. Although the JBrowse portion of that task is essentially complete, the website work at ZFIN remains to be completed. We also have early development of FlyBase and SGD JBrowse instances. The remaining member MODs are in the planning stage of migrating their JBrowse to the Alliance infrastructure.

#### Genes

**Fig. 3.**
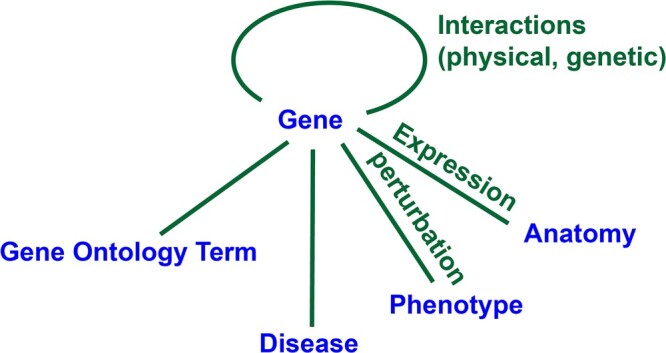
Conceptual map of gene-centered information. Perturbations of gene activity include alleles, variants, RNAi, knockdown, and transgenic overexpression.

Information about genes is core to the Alliance, and thus the first major type of reports (or webpages) is for genes. Genes are connected to a rich set of information (Fig. 3).

### Summary of data included in the Alliance

Through years of biocuration at the individual MODs, assertions have been carefully added to knowledge bases. Table 1 lists a sample of the entities and assertions included in the Alliance.

**Table 1.** Some of the entities or data types and numbers of objects in the Alliance Central Portal.

Entity or data type	Number
Species	8
Gene	291,439
Synonym, identifier	1,341,412
Association, phenotype	1,799,889
Association, gene expression	1,579,792
Association, gene-disease	233,772
Gene–gene genetic interactions	635,565
Gene–gene physical interactions	1,826,673
High-throughput (HTP) dataset samples	229,581
Variant protein sequence	218,097
Alleles and variants	404,596,017
Genomic locations	8,506,484
Constructs	195,753
Publications	222,671
Gene ontology (GO) annotations	1,792,808
Fly anatomy ontology (FBbt) terms	17,475
Worm anatomy ontology (WBbt) terms	7,192
Mammalian phenotype ontology (MP) terms	13,752
Zebrafish experimental conditions ontology (ZECO) terms	161
Genomic locations	8,506,484
Exons	3,549,356

A complete list of ontologies used by the Alliance can be found at https://www.alliancegenome.org/privacy-warranty-licensing#ontology [accessed 2022 Jan 16].

#### Examples of curated statements

Curators vet and bring in knowledge in the form of assertions, that is, statements relating to entities. For example, a gene expression statement is of the form: *Gene A is expressed in body part B based on method C according to reference D*. A variant statement is of the form: *Variant A was constructed by Method B and has sequence change C*. Phenotype statements are of the forms: *Variant A results in Phenotype B* or *Overexpression of Gene A by Construct B results in Phenotype C*. Much of the curation involves defining the appropriate entities, their relationships, and referenced type of evidence.

### Orthology

Orthology assertions are key to comparative genomics. The Alliance has standardized the ortholog calls across the model organisms and human so that the user obtains the same orthologs regardless of starting point. The orthology assertions are based on the combination of a set of state-of-the-art algorithms sanctioned by the Quest for Orthologs Consortium (Linard *et al.* 2021), using the DIOPT method (Hu *et al.* 2011). The assertions are by no means complete but they are consistent. Omissions will be obvious and help improve the algorithms or set of assertions. For example, the calls do not include hand-done analyses such as iterative approaches like HMMer that do not seem to be automatable, and this is an active area of research (e.g. Martín-Durán *et al.* 2017; Weisman *et al.* 2020), As a case in point, *C. elegans affl-2* can be considered orthologous to the human *AF4/FMR2* family proteins based on JackHMMer (Walton *et al.* 2020) but is not called by the Alliance, presumably due to its low level of similarity and the distribution of conserved residues across the protein such that it is missed by multiple alignments; also, ZFIN hand curates orthologs that are not called by the Alliance.

Orthology can be used to fill in missing information about gene function, accepting by default the “ortholog conjecture” that orthologs have the same function. For example, subcellular localization might be known from 1 organism, and phenotype-based inference of function from another.

### Gene function

The Alliance is helping develop infrastructure for GO curation and display. Connection of gene products to GO terms describing the molecular activity, localization, and broader biological process has long been a significant biocuration task. Curation of experimentally tractable research organisms with GO can be propagated phylogenetically to organisms such as human (Gaudet *et al.* 2011), providing insight into the functions of human genes and elucidating experimental data from both human and research organisms through gene set enrichment analyses.

Any gene can have a large number of GO terms associated with it, reflecting multifunctionality and the fact that the same module can be repurposed in different contexts. We provide a number of ways to provide Alliance users with a more intuitive picture of a gene’s function. First, we use algorithmic methods to distill the many terms used to annotate a gene into a textual gene description. Second, we developed a visualization called GO ribbons that provides a visual summary of the function of a gene (or a group of orthologous genes defined by DIOPT) summed up to higher level terms. Third, we make use of our next-generation of GO annotations, called GO-CAMs (Causal Activity Models; Thomas *et al.* 2019). These GO-CAMs provide a contextualized view of gene function, where the function of a gene can be explored in the context of the function of interacting genes or genes in the same pathway (see Fig. 7).

### Gene expression

**Fig. 4.**
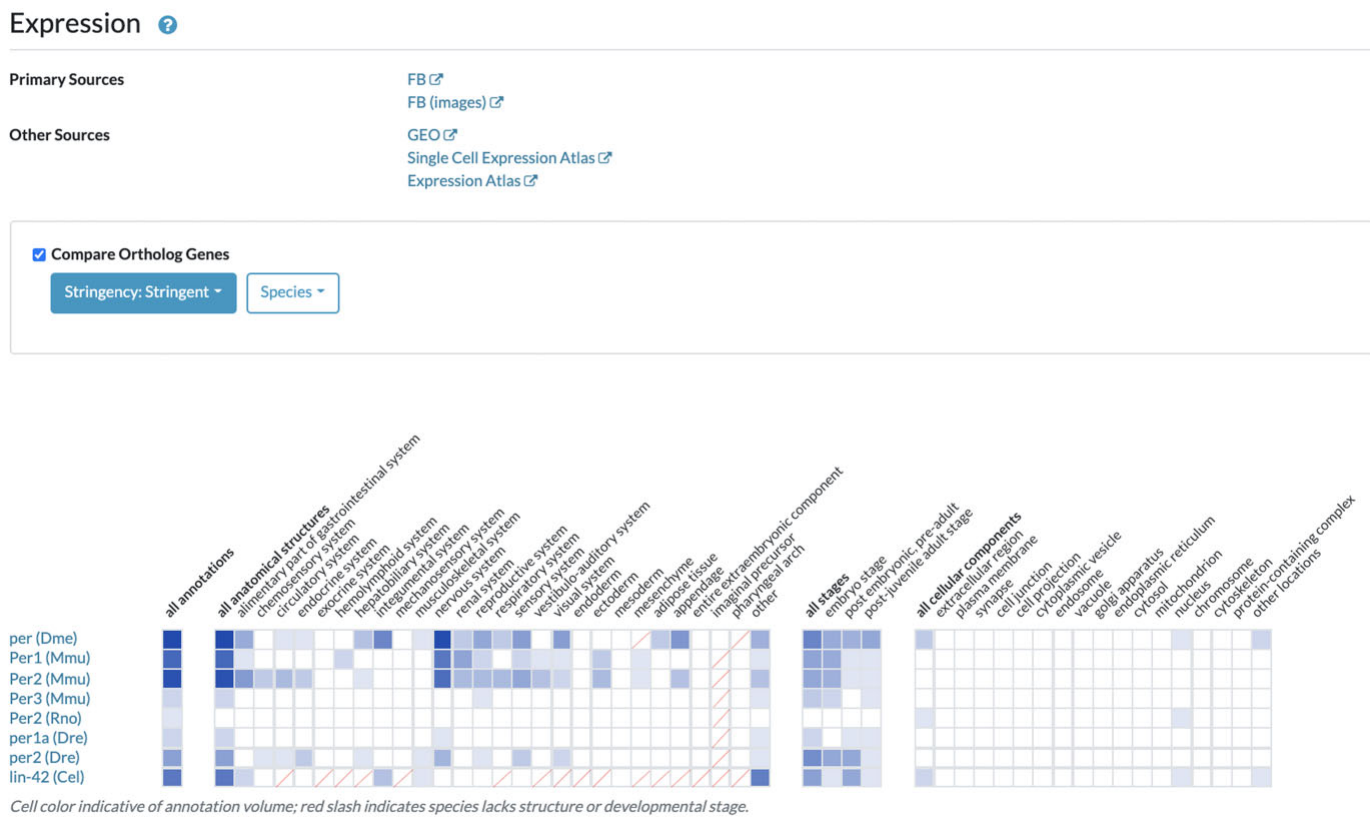
The expression widget. This example is for the *Drosophila* gene *per*. The links to primary sources are customized and the number varies among species depending on data.

An integrated view of wild-type expression data can be accessed via the Expression widget. The widget contains a gene expression ribbon that summarizes spatio-temporal localization and displays subsections for anatomical location, developmental stage, and subcellular location. Core metadata of the annotations are captured using the relevant bio-ontologies. To improve readability, UBERON terms (Haendel *et al.* 2014), to which model organism anatomy and stage ontology terms are mapped, were selected for the high-level anatomical structures and developmental stages ribbons. The cellular component section displays a carefully designed subset of GO Cellular Component terms, consistent with the corresponding ribbon in the Function section. Ribbon boxes are shaded when annotations are present, and the color intensity represents the number of expression annotations, with darker hues indicating more data (Fig. 4). Red lines/slashes across a box indicate that the term is not appropriate for an organism. Clicking on a shaded box produces a data table showing additional details for the annotation, such as the assay, the original publication from which data have been curated and links to the original data at individual MODs. Data can be sorted by any of the values and downloaded as a tab-delimited file by clicking the “Download” button below the table.

In addition to displaying gene expression data for individual species, users can compare gene expression data across species by selecting the ‘Compare Ortholog Genes’ checkbox at the top of the ribbon. When the orthology picker is selected, expression data for orthologous genes are added to the ribbon summary and the data table, when present (Fig. 4). The Expression widget also contains hyperlinks to primary sources of annotated data, e.g. the MODs, and external sources of gene expression data, such as Gene Expression Omnibus (Clough and Barrett 2016https://www.ncbi.nlm.nih.gov/geo/ [accessed 2022 Jan 16]); the Expression Atlas (Papatheodorou *et al.* 2020, https://www.ebi.ac.uk/gxa/home [accessed 2022 Jan 16]), and the Single Cell Expression Atlas (Moreno *et al.* 2022, https://www.ebi.ac.uk/gxa/sc/home [accessed 2022 Jan 16]). Expression data can also be downloaded in bulk for all organisms or for individual species on the Data Download page (https://www.alliancegenome.org/downloads [accessed 2022 Jan 16]).

We recently imported MOD-curated metadata for high-throughput (HTP) (RNA-seq and microarray) gene expression studies and made them available for searching on the Alliance website. To browse HTP metadata at the Alliance, one can select the “HTP Dataset Index” category by clicking “All” in the Search box. Results can be further narrowed using standardized metadata annotations done by Alliance curators; these include: species, tags, assays, tissues, and sex. Search results link back to the individual MODs or Gene Expression Omnibus (GEO).

Planned future improvements include completing data harmonization of the classes used in the expression annotation model, such as images, movies, and molecular reagents; the inclusion of expression in nonwild-type backgrounds; and annotations of absent or ambiguous tissue expression. The implementation of a content-rich expression summary page will provide a unified way to access all expression data associated with a specific gene.

### Disease and phenotypes

The Alliance links phenotypes and human diseases to genes, alleles, genotypes, and strains. Harmonized disease and phenotype data from the source MODs are displayed on gene, allele, and disease pages.

We have expanded the types of information associated with disease and phenotype annotations to provide greater functionality. This involved harmonizing new associated information types from the source MODs, implementing the display of new details associated with relevant entities, and improving the display of existing data. During the past year, we have harmonized our representation of transgenic alleles by creating constructs as a new entity and associating these with alleles. Constructs include information about expressed genes, regulatory regions, RNAi targets for knock-ins, and transgenic alleles. Because the expressed genes in constructs are connected to the species of origin of the gene, transgenic allele data are now displayed in a new section on species-specific gene pages for the gene that is expressed. For example, the human APP gene page lists transgenic alleles expressing human APP in fly and mouse. These transgenic alleles are in some cases used to test conserved function of orthologs, in other cases the way in which a gene was identified, as disease models, or humanized model organism*s*.

A major expansion of information for phenotype and human disease annotations is the harmonization and integration of experimental conditions. The experimental conditions incorporate chemical, dietary, and physical interventions used to induce and/or modify phenotypes and human disease models. The experimental conditions make use of a number of ontologies and controlled vocabularies, including ChEBI, ZECO, and XCO. A set of high-level terms from ZECO are used to group similar types of conditions (e.g. chemical treatment). Annotations including experimental conditions can now be seen on gene, allele, and human disease pages. For example, the disease page for Parkinson's disease (DOID: 14330) now includes zebrafish and worm models generated using “chemical treatment: Oxidopamine.” Tables on the pages can be sorted and filtered using the experimental conditions, and this information is included in the download files.

### Variants

The incorporation and presentation of variants is a high priority for the Alliance. The focus of recent work has been to improve the display of manually curated variants associated with phenotypic alleles and to incorporate a large corpus of HTP variants from large-scale sequencing efforts for all Alliance species, including human. To this end, model organism HTP variants are submitted by Alliance members (FlyBase, RGD, SGD, WormBase) or directly imported from EVA (mouse and zebrafish). Human variants are imported from Ensembl (Cunningham *et al.* 2021). Alleles, allele-associated low-throughput variants, and HTP variants are all searchable through the Alliance general search and can be explored using the Allele/Variant filters in the left sidebar of the search results page.

**Fig. 5.**
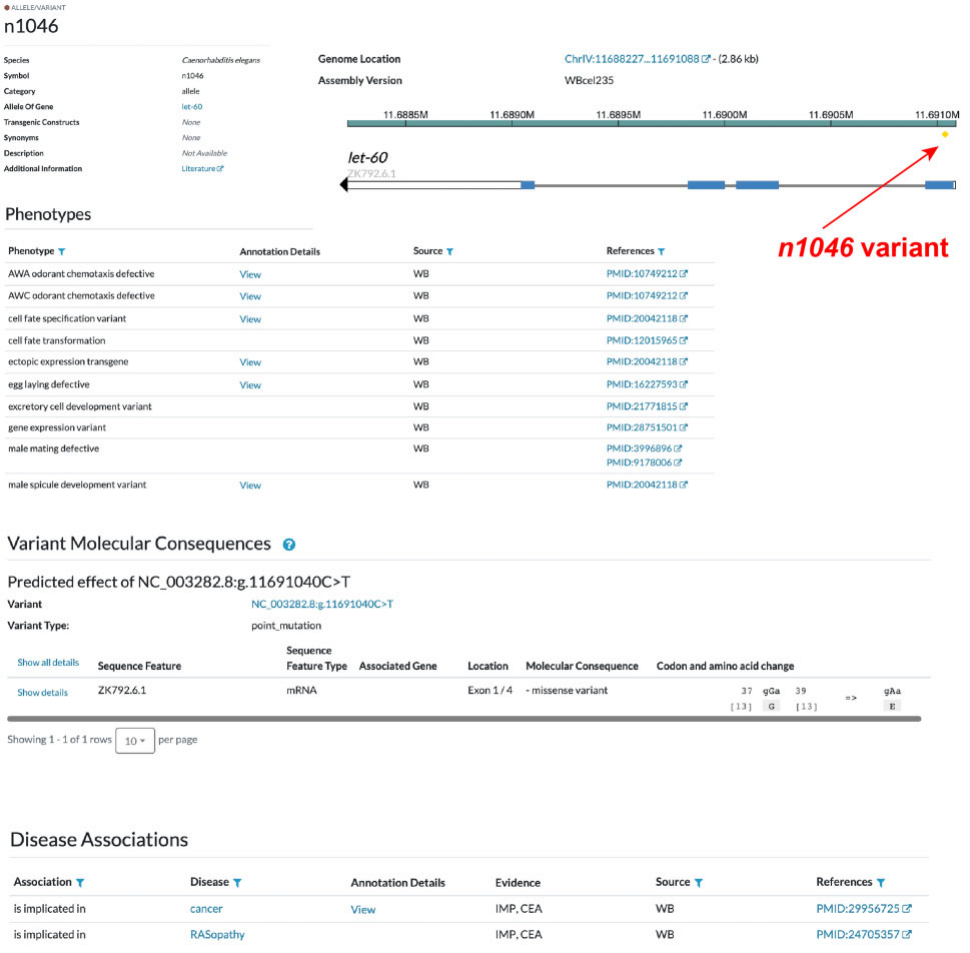
Montage of types of variant information and displays. The variant page has a summary, snapshot of Genomic Location, and then tables of Phenotypes, Molecular Consequences, and Disease Associations.

During the past year, we enhanced the display of variant information on the gene page and on the allele/variant page (Fig. 5). On both pages, an interactive Sequence Viewer shows the low-throughput variant(s) in the context of the gene and its associated transcripts. When a variant is selected in the viewer, a popup with details about that variant is displayed. On the gene page, that variant is also highlighted both in the viewer and in the allele/variant table below. Conversely, selecting a variant in the table highlights it in the viewer.

The allele/variant table on the gene page now lists all alleles and variants, including HTP variants, associated with the gene. By default, the alleles of the gene are listed first with information about their associated variants when those data are available, followed by the list of all HTP variants that overlap the gene. For each entry, the category [i.e. allele, allele with associated variant(s), or variant] is provided. For allele-associated and HTP variants, the genomic position and nucleotide change is stated in HGVS nomenclature, the variant type is listed, and molecular consequences for that variant are listed. Disease and/or phenotype annotations are provided for alleles, when known.

Below the table, a button labeled “View detailed Alleles/Variants information” leads to a newly created “alleles/variants details” page. This page presents the low-throughput variants in their gene-level context in the same sequence viewer display as found on the gene page. An expanded table here includes all of the alleles and variants for the gene with specific information about the molecular consequences of each variant on each associated transcript. Our newly instituted variant annotation pipeline takes variant data from the Alliance, runs the Ensembl Variant Effect Predictor tool (McLaren *et al.* 2016), and returns the variant type, predicted consequences, and HGVS nomenclature for each. Consequences of missense variants are further annotated with predicted pathogenicity using Polyphen-2 (Adzhubei *et al.* 2013) and SIFT (Kumar *et al.* 2009). When a variant overlaps more than 1 gene, the details table includes consequences for that variant for all the overlapping transcripts. All variant information (including the variant specifications and the effects of the variant on each transcript) is available for download both from Alliance report pages (gene, allele/variant, alleles/variants details) and from the Alliance Downloads page.

### Automatically generated concise gene summaries

**Fig. 6.**
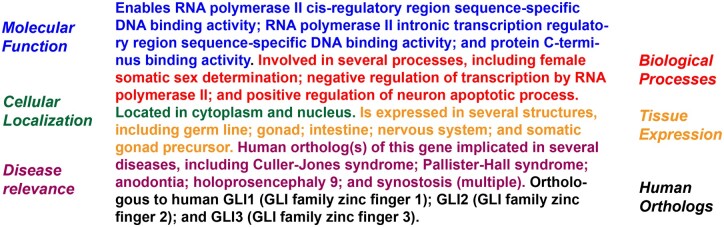
Automatically generated gene summaries from structured data. Example of a gene summary for *C. elegans* gene *tra-1* showing different data categories highlighted in different colors.

With each new Alliance release we automatically generate short human-readable gene summaries for the 6 model organism species and human (Kishore *et al.* 2020). These text summaries are displayed in the top section of Alliance gene pages and describe a gene’s function, molecular identity, the biological processes it participates in, its expression and activity in cellular components and tissues, and its relevance to human disease (Fig. 6). Updates were made recently so that the gene summaries algorithm uses the GO annotation file (GAF) 2.2 format, specifically to include the relation between a gene product and GO term. The inclusion of these relations provides more nuanced statements that describe a gene, such as “acts upstream of” (a biological process), and “located in” (a cellular component) (http://geneontology.org/docs/go-annotation-file-gaf-format-2.2/#qualifier-column-4 [accessed 2022 Jan 16]).

The Alliance 5.0.0 release has more than 122,000 gene summaries across all of the Alliance species. These summaries are also available for download from the Alliance “Data Downloads” page under the “Gene Descriptions” section (https://www.alliancegenome.org/downloads [accessed 2022 Jan 16]) and also via the Alliance data Application Programming Interface (API) (https://www.alliancegenome.org/api/swagger-ui/ [accessed 2022 Jan 16]) under the “Genes” endpoints.

### Interactions

Examining the interactions between genes can be crucial to deducing their function. A set of interactions (a “hairball” graph with genes as nodes and interactions as edges) provides some clues and helps predict which genes are worth studying further. We thus seek to display a comprehensive set of interactions linked to our other data. Two major types of interactions are molecular interactions, which indicate proximity and often direct physical contact of their products, and genetic interactions, which indicate functional connections. Because molecular interactions do not necessarily imply common function, and a genetic interaction does not necessarily imply physical interaction, we include both.

#### Molecular interactions

We continue to provide annotations of molecular interactions (e.g. protein–protein and protein–DNA interactions) between genes and gene products for the current 7 Alliance species, including humans, on Alliance gene pages, downloadable molecular interactions files on the Alliance Downloads page, and programmatic access to molecular interaction data via APIs. During the past year, in an effort to help Alliance users discover information pertinent to the ongoing COVID-19 pandemic, we imported human-SARS-CoV-2 virus protein–protein interactions into the Alliance from the BioGRID interaction database (https://thebiogrid.org/ [accessed 2022 Jan 16]; Oughtred *et al.* 2021) and IMEx consortium (https://www.imexconsortium.org/ [accessed 2022 Jan 16]; Orchard *et al.* 2012), making these interactions available on respective human gene pages as well as on newly developed SARS-CoV-2 virus gene pages. These new SARS-CoV-2 gene pages provide users with basic gene information (IDs, names, aliases, cross-references, etc.), links to a dedicated SARS-CoV-2 JBrowse instance, and molecular interactions with human proteins. A list of human proteins that have been found to interact with SARS-CoV-2 virus proteins is now provided on the Alliance coronavirus resources page (https://www.alliancegenome.org/coronavirus-resources [accessed 2022 Jan 16]).

#### Genetic interactions

Genetic interactions, for example phenotypic suppression, represent evidence of functional interaction (direct or indirect) between genes involved in the same biological processes. We now provide genetic interaction annotations for Alliance genes on gene pages, downloadable interactions files on the Alliance Downloads page, and via our APIs. The gene page Genetic Interactions table provides the identity of genes that interact genetically with the focus gene (the gene whose page a user is currently viewing) along with the roles of each interacting gene (e.g. suppressor/suppressed), each gene’s genetic perturbation (e.g. suppressing/suppressed mutations, if available), the genetic interaction type, the phenotype or trait affected, the source of the annotation (with hyperlinks), and the references in which the genetic interaction was reported (with hyperlinks to PubMed). Descriptions of genetic interactor roles and genetic interaction types from the PSI-MI controlled vocabulary (Kerrien *et al.* 2007) are available to users as tooltip pop-ups when hovering the cursor over a term name in the gene page table. A download option is provided to download the gene’s genetic interaction data (incorporating sorting and filter options). These genetic interaction data are sourced from Alliance members WormBase and FlyBase as well as from BioGRID, together constituting a complete set of the curated interactions.

### Pathways

**Fig. 7.**
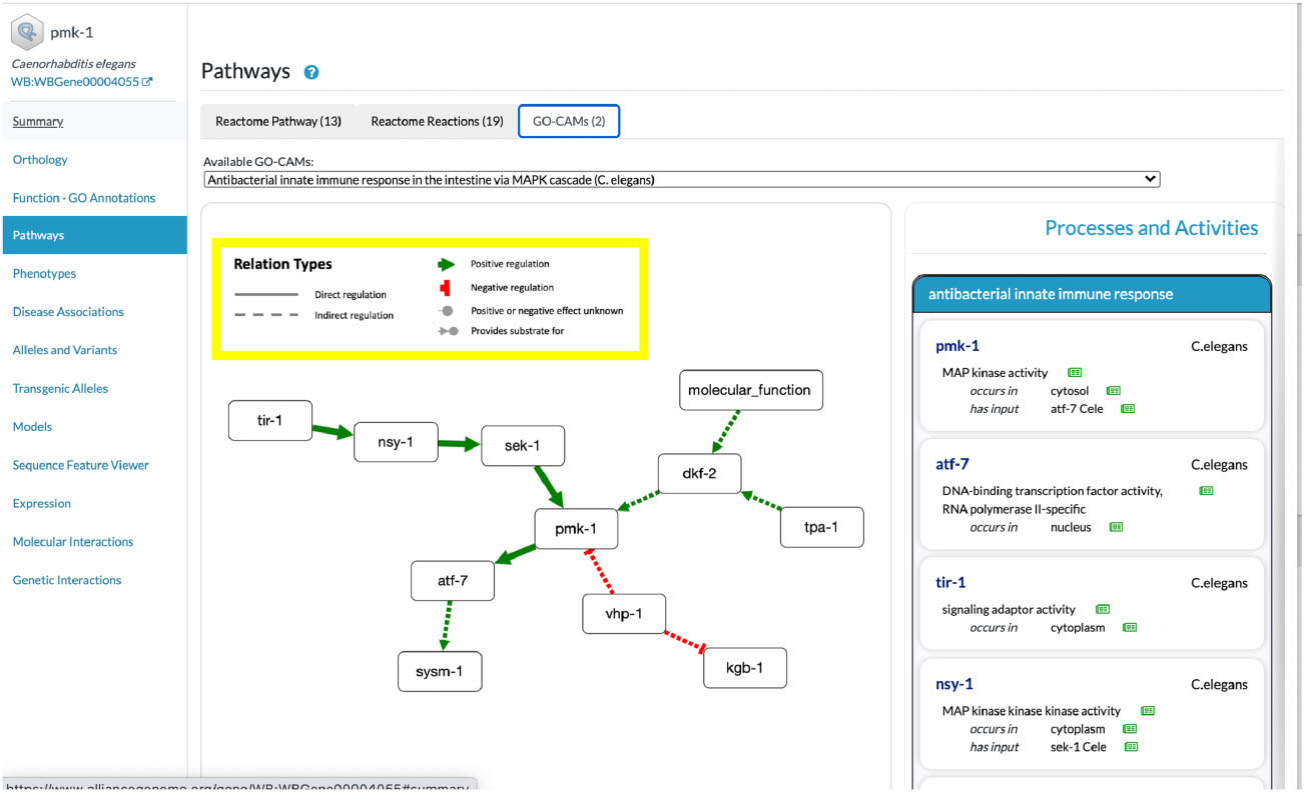
Views of pathways. GO-CAM model with simplified view for *pmk-1*.

We chose to employ 2 widely used systems to model pathways, Reactome and GO-CAM. Work to harmonize these representations has been done by the GO Resource (Thomas *et al.* 2019; Good *et al.* 2021). We then developed a pathway widget for Alliance gene pages (Fig. 7). This includes manually curated human Reactome pathways and their corresponding pathways for other organisms mapped via orthology, and manually curated GO-CAM pathways.

### Search portal

Information about 1 research organism is daunting, and with 7 (and more in the future), an effective search tool is crucial. At the top right corner of every Alliance page is a search box that provides an entry point into Alliance data. Typing into the search box brings up autocomplete suggestions that offer direct links to specific Alliance pages. For example, typing “pten” into the box brings up a list of PTEN genes from various species; clicking on a suggestion opens that page. If a suggestion is not selected, the search tool returns the broadest possible set of results, with the most relevant results sorted at the top, and filters that provide further refinement of results based on various types of data associated with those search results. Several principles guided the development of this search tool.

First, the search tool must provide users with enough information to evaluate the quality and relevance of the results. This is accomplished, in part, by providing a succinct summary for each search result; gene results, for instance, list the accepted symbol, various synonyms, and a gene synopsis so that the gene’s identity is apparent. In addition, the results highlight the information used in the result. Second, the search tool must return results that are indirectly related to query terms by using well-established relationships between database objects. For example, the search tool is “aware” of relationships between ontology terms, such that a search for “cell-cell junction” returns matches to the more specific term “gap junction,” or a search for “eye” returns matches to “retina,” and so forth. Similarly, ortholog data are included in the search. Third, the search tool must make the best results easy to find by sorting them to the top of the results page, using a calculated relevance score. For example, human genes are scored higher than model organism genes, and protein-coding genes are scored higher than pseudogenes. Fourth, the search tool has filters that support refinement of the results. Each filter represents a distinct data type (e.g. disease) and is populated with terms associated with annotations for that data type (e.g. high level disease terms like “monogenic disease” or “cancer”) as well as the number of results associated with that term. Finally, the search returns “related data links” that provide quick retrieval of related objects from different data categories, and data can be browsed using the search tool if no search term is provided.

### AllianceMine

To facilitate more complex and diverse needs of researchers to search and compare data across different organisms, most of the Alliance MODs have InterMine instances. We reasoned that a central instance would save effort on maintenance and expansion, because it can be built on the harmonized data in the Alliance Central infrastructure. The Alliance has thus implemented InterMine (http://intermine.org/), an open-source data warehouse system that comes out of the box with a sophisticated querying interface. InterMine is a widely used data mining tool that builds a database by loading various data types into a single data warehouse that enables queries as though the data were merged.

**Fig. 8.**
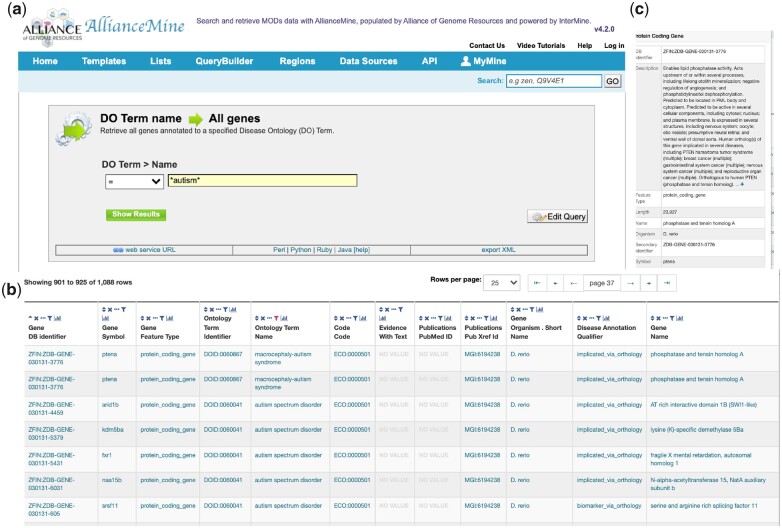
AllianceMine. Screen shots of AllianceMine output. Using a template query of disease ontology (DO) to all genes with the term “autism” a) returns 1088 genes b). Mousing over *petena* pops up a brief description of that gene c).

AllianceMine (https://www.alliancegenome.org/alliancemine/ [accessed 2022 Jan 16], Fig. 8) provides an advanced search and analysis tool to query harmonized data. It is quite multifaceted in that a search can be initiated with a single gene or a list of genes, a list of Gene Ontology terms or other data types. By utilizing the List functionality users can ask advanced biological questions and get answers by List manipulation. In addition to being a scratch interface, it can also act as a discovery tool, a curation aid, and a quality control tool.

AllianceMine currently has the following data types: Chromosomes, Genes, GO, Disease, Alleles/Variants, and Orthology. We will continue to add new data types in future releases. Intermine requires continual updating as additional data are added, and maintenance takes considerable energy.

### Community support by the Alliance

We made 2 improvements to our community support. Each MOD has some type of community forum (message board, etc.). As a step toward streamlined support for common functions, we implemented a common forum using the platform Discourse. The slight disadvantage of an extra click to get to the organism of choice is offset by access to responses to generic questions that arise in the context of 1 research organism but apply broadly to others, e.g. polymerase chain reaction (PCR), bioinformatics, sources of reagents, puzzles about genetics, physiology, evolution, and so forth.

We have put in place a simple yet effective mechanism for user feedback where users can email the Alliance for assistance; these emails are directly integrated into our Jira issue tracking system. We also maintain a presence on Twitter (https://twitter.com/alliancegenome [accessed 2022 Jan 16]).

### Biocuration

Information about each organism in a knowledge base is there because of curation, the process of choosing which information to include and standardizing its format using unique identifiers, often including metadata (e.g. what sample a transcriptome analysis comes from). Curation requires knowledge of the data and how they are described, the standard vocabularies or ontologies used, and the data model (database schema) that allows the information to be stored, transformed, and accessed. As such, biocuration is the major task of the individual MODs, each of which has built impressive but idiosyncratic workflows and software infrastructure for their curation. There is a continual quest for increased efficiency (and accuracy) and thus development of methods and software. At a granular level, effective autocomplete for terms saves time and decreases mistakes and tedium. At higher levels, improvements to curation have not, in general, propagated across MODs, likely because of the high technical barrier to redeployment in very different workflows. These improvements include use of machine learning (ML), artificial intelligence (AI), and text mining to speed up the curation workflow. Realizing this, the Alliance has started to build a common curation system, using knowledge (and software components) from the broader biocuration community. Another aspect of curation is obtained from authors and the community the MODs serve. Curators often contact authors directly for clarification or missing datasets. Systematic calls for help, such as defining the information present in a given paper, are made by SGD, FlyBase, and WormBase. Such curation requires workflows and software honed by experience, and the common system can allow hard-learned lessons to be applied across communities.

Besides curation, the Alliance will use its literature system to link genes, anatomy terms, variants, transgenes, antibodies, pathways, and diseases to specific papers.

#### Literature acquisition

Literature acquisition was a natural starting point for building common infrastructure and interfaces. Previously, MODs have developed their own tools and workflows to deal individually with the task of finding and acquiring publications to curate. These separate efforts can be supported at the Alliance, taking the best aspects of each system and supporting each MOD’s curation efforts while reducing overall maintenance and overhead costs. This system will consist of a database, APIs, an editorial interface, and workflow tracking.

We have been developing a persistent database to store publication records and create a combined library of resources. This database will serve as an incoming port for PubMed articles, which is the primary source for all Alliance references with PMIDs. In addition, it will contain a resource editing UI for members of the Alliance to enter publication records that are not indexed by PubMed or MOD-specific resource, such as theses, meeting abstracts, personal communication, and Alliance group curation efforts. This UI will also facilitate search and editing of metadata for any reference in the database.

The UI, under development, contains an editing interface that will allow us to continue to deal with these and other discrepancies that are found when loading publications. In addition, it will allow manual entry of unique IDs (PMIDs, DOIs, and MOD-specific identifiers) and associated bibliographic information when needed, such as for nonPubMed papers.

An end goal of establishing this persistent library of resources is to utilize common curation forms for the harmonized curation schemas developed by other working groups in the Alliance. APIs will be used to allow access to the Alliance Library for MOD-agnostic data display, data retrieval, and curation data capture.

Every paper in the system will be marked as to whether it belongs in a particular MOD’s corpus. Papers may not belong in a particular corpus if the subject is not the model organism but describes a protocol or method, for example. History tracking has been instantiated in the database so we can see when a paper was entered, how it was entered, which keywords were found, and when changes were made. Toward this end we are also working on a shared login system so we will know which MOD is involved when curators are working on a paper. We will extend this system to send papers to the correct MOD for further curation.

In the future, we will work on integrating the literature database with natural language processing (NLP) and other computational pipelines, some of which are already employed by member databases such as FlyBase, RGD, and WormBase (Müller *et al.* 2004, 2018; Van Auken *et al.* 2009; Rangarajan *et al.* 2011; Fang *et al.* 2012; Liu *et al.* 2015; Arnaboldi *et al.* 2020; Hu *et al.* 2020). We will start testing with the full text of papers available at PMC so that algorithms can be optimized. When these pipelines are in place, the literature database will need to be expanded to encompass the computationally associated metadata.

### Prospects

The Alliance has already provided new features for all communities. In particular, we provide comparative views in the form of Ribbons, and variant effect predictions. We also added concise gene descriptions for zebrafish and standardized all existing gene descriptions. We are *en route* to provide centralized InterMine and JBrowse instances as well as a standard literature service. We will soon start developing a shared BLAST-like service at the Alliance, one that will serve the needs of both existing MODs as well as the future needs of the Alliance.

We have a plan to include support for paralogs within a species (“in-paralogs”), with comparison ribbons for Gene expression, phenotypes and GO terms. We will include links to key reagents, such as Gal4 driver lines, strains, fish, plasmids, and so forth.

A definite challenge facing the Alliance is how to deal with the many features that each community is used to having. We believe we can include much of the long tail of features (referring to the distribution of the number of groups that have each feature). Although the goal is to be the union of features, it will take a while to generalize each feature. We thus have plans to bring in (or link to) MOD-specific data and displays. In this way, we will gradually increase services for all while not losing anything useful.

We have designed but not yet implemented organism/community-specific landing pages. These are a first step toward supporting individual communities in the Alliance infrastructure. These pages will replace the home pages of individual resources and retain much of the same functionality. Another customization will be organism-specific data, displays, and tools. The data will be displayed on the relevant report page. For example, a *C. elegans-*specific gene expression dataset will be displayed in the gene expression section or a special gene expression page.

The Alliance is now poised to bring in other communities. A mature model organism knowledgebase, Xenbase, which focuses on the tetraploid *Xenopus laevis* and diploid *Xenopus tropicalis*, has begun to participate in the Alliance. They are harmonizing some of their key data and are working with the Quest for Orthologs and DIOPT to establish standard orthology information, at which time we can generate gene pages. We therefore explore integrating Xenbase as a test of our infrastructure and processes and to provide useful service to additional communities.

## Data availability

Data are available by browsing, displays analytical tools, downloads, and APIs via the Portal at alliancegenome.org.
